# Investigation of the *HSPG2* Gene in Tardive Dyskinesia – New Data and Meta-Analysis

**DOI:** 10.3389/fphar.2018.00974

**Published:** 2018-09-18

**Authors:** Clement C. Zai, Frankie H. Lee, Arun K. Tiwari, Justin Y. Lu, Vincenzo de Luca, Miriam S. Maes, Deanna Herbert, Anashe Shahmirian, Sheraz Y. Cheema, Gwyneth C. Zai, Anupama Atukuri, Michael Sherman, Sajid A. Shaikh, Maria Tampakeras, Natalie Freeman, Nicole King, Daniel J. Müller, Lior Greenbaum, Bernard Lerer, Aristotle N. Voineskos, Steven G. Potkin, Jeffrey A. Lieberman, Herbert Y. Meltzer, Gary Remington, James L. Kennedy

**Affiliations:** ^1^Tanenbaum Centre for Pharmacogenetics, Molecular Brain Science, Campbell Family Mental Health Research Institute, Centre for Addiction and Mental Health, Toronto, ON, Canada; ^2^Department of Psychiatry, University of Toronto, Toronto, ON, Canada; ^3^Laboratory Medicine and Pathobiology, University of Toronto, Toronto, ON, Canada; ^4^Institute of Medical Science, University of Toronto, Toronto, ON, Canada; ^5^The Danek Gertner Institute of Human Genetics, Sheba Medical Center, Tel Hashomer, Israel; ^6^The Joseph Sagol Neuroscience Center, Sheba Medical Center, Tel Hashomer, Israel; ^7^Sackler Faculty of Medicine, Tel Aviv University, Tel Aviv, Israel; ^8^Biological Psychiatry Laboratory and Hadassah BrainLabs, Hadassah-Hebrew University Medical Center, Jerusalem, Israel; ^9^Department of Psychiatry and Human Behavior, Long Beach Veterans Administration Health Care System, University of California, Irvine, Irvine, CA, United States; ^10^Columbia University, New York State Psychiatric Institute, New York City, NY, United States; ^11^Psychiatry and Behavioral Sciences, Pharmacology and Physiology, Chemistry of Life Processes Institute, Northwestern University Feinberg School of Medicine, Chicago, IL, United States

**Keywords:** pharmacogenetics, tardive dyskinesia, schizophrenia, perlecan/heparan sulfate proteoglycan 2 (HSPG2), meta-analysis

## Abstract

Tardive dyskinesia (TD) is a movement disorder that may occur after extended use of antipsychotic medications. The etiopathophysiology is unclear; however, genetic factors play an important role. The Perlecan (*HSPG2*) gene was found to be significantly associated with TD in Japanese schizophrenia patients, and this association was subsequently replicated by an independent research group. To add to the evidence for this gene in TD, we conducted a meta-analysis specific to the relationship of *HSPG2* rs2445142 with TD occurrence, while also adding our unpublished genotype data. Overall, we found a significant association of the G allele with TD occurrence (*p* = 0.0001); however, much of the effect appeared to originate from the discovery dataset. Nonetheless, most study samples exhibit the same trend of association with TD for the G allele. Our findings encourage further genetic and molecular studies of HSPG2 in TD.

## Introduction

Schizophrenia is a serious long-term psychiatric disorder treated primarily with antipsychotic drugs. However, each intervention has side effects and in the case of typical antipsychotics, such as haloperidol and perphenazine, tardive dyskinesia (TD) is one of them. TD is a severe, potentially irreversible movement disorder that is characterized by athetoid movements that affect mainly the mouth, tongue, jaw, and muscles for facial expressions; it may also affect the upper limbs, lower limbs, neck and trunk. These movements may persist even after medication has been withdrawn and can worsen with age (Solmi et al., 2018). Between 20 and 25% of chronic schizophrenia patients treated with first-generation antipsychotics develop TD (Margolese et al., 2005; Tarsy and Baldessarini, 2006). The risk of TD has been associated with older age, female sex, longer duration of antipsychotic treatment, and use of older, typical, first-generation antipsychotics such as haloperidol, perphenazine, and chlorpromazine (Martino et al., 2018). Atypical antipsychotics, such as clozapine, olanzapine, and quetiapine, are associated with a lower risk of developing TD (Solmi et al., 2018). While TD rates have declined with increased use of atypical antipsychotics, TD risk has not been completely eliminated (Correll and Schenk, 2008).

The cause of TD remains unclear. The mechanism of TD development has been hypothesized to involve supersensitivity of the nigrostriatal dopaminergic pathway, damage to neurons by free radical overproduction, and dysregulation of the GABAergic system (Lee and Kang, 2011). Familial occurrence of TD also supports a genetic component in TD (Weinhold et al., 1981; Yassa and Ananth, 1981; Müller et al., 2001). As such, a number of findings have emerged from candidate gene studies (Zai et al., 2018a). For example, the dopamine D2 receptor (*DRD2)* gene has been a primary candidate (Zai et al., 2007a; Bakker et al., 2008). More recently, there is growing evidence that the vesicular monoamine transporter 2 (VMAT2/SLC18A2) gene may be associated with TD, including association findings from genetic studies (Tsai et al., 2010; Zai et al., 2013) and promising findings from clinical trials on the VMAT2 inhibitors deutetrabenazine and valbenazine as treatment for TD (Anderson et al., 2017; Factor et al., 2017; Fernandez et al., 2017; Hauser et al., 2017). However, the associated genetic markers had only moderate effect sizes, suggesting additional genetic factors likely contribute to TD susceptibility. A number of genome-wide association studies (GWASs) of TD have been conducted (Aberg et al., 2010; Greenbaum et al., 2010; Syu et al., 2010), leading to a number of novel candidate genes, including the Perlecan-coding gene *HSPG2* (also known as Heparan Sulfate Proteoglycan 2; HGNC ID: 5273; at 1p36.12).

The *HSPG2* rs2445142 G allele was found to be the risk allele for TD in a GWAS on 86 Japanese schizophrenia patients with treatment-resistant TD and 186 without. This allele was also associated with an increase in gene expression in human prefrontal cortical tissues (Syu et al., 2010). Administrations of the typical antipsychotic haloperidol in mice increased HSPG2 expression after 4 weeks, but decreased expression after 50 weeks (Syu et al., 2010). More importantly, *Hspg2* deficiency led to fewer vacuous chewing movements in a mouse model of TD (Syu et al., 2010). The *HSPG2* association was replicated in a refined sample from the CATIE trial, consisting of 179 schizophrenia patients of European ethnicity (Greenbaum et al., 2012), as well as a sample of Jewish Israeli schizophrenia patients (Greenbaum et al., 2012). Thus, to further validate these findings, we conducted an association study of *HSPG2* rs2445142 with TD and followed up with a meta-analysis.

## Materials and Methods

### Subjects

For this meta-analysis, we added two datasets to the currently available data from the literature. The first dataset included 217 participants from two samples (Canada, United States) for which the sample characteristics have been described previously (Basile et al., 1999; Zai et al., 2007b, 2017). Briefly, participants were enrolled from one site in Canada and three sites in the United States: Center for Addiction and Mental Health in Toronto, Toronto, ON, Canada (Dr. G. Remington, *N* = 94); Case Western Reserve University in Cleveland, Cleveland, OH, United States (Dr. H. Y. Meltzer, *N* = 63); Hillside Hospital in Glen Oaks, Glen Oaks, NY, United States (Dr. J. A. Lieberman, *N* = 48); and University of California at Irvine, Irvine, CA, United States (Dr. S. G. Potkin, *N* = 12). Participants had DSM-III-R or DSM-IV diagnoses of schizophrenia or schizoaffective disorder (American Psychiatric Association, 2000); individuals with type II diabetes, head injury with loss of consciousness, or seizure disorder were excluded from the study. Patients recruited in the United States (HYM, JAL, SGP) had no prior exposure to atypical antipsychotics, while the chronic patients from Canada (GR) may have been on either typical or atypical antipsychotics. Overall, all patients had been exposed to typical antipsychotic medication for at least 1 year before TD assessment. The rate of TD was not significantly different between the United States (38%) and Canadian (43%) samples (*p* = 0.58), and was lower, albeit not significantly, in males (36%) versus females (49%) in the collective sample (*p* = 0.11).

Our second dataset consisted of schizophrenia or schizoaffective disorder patients from a naturalistic pharmacogenetic study [The Individualized Medicine: Pharmacogenetics Assessment and Clinical Treatment (IMPACT, *N* = 20 TD cases and 41 TD-negative controls at baseline)] (Herbert et al., 2017).

The classification of TD was based on the Schooler and Kane criteria, using the Abnormal Involuntary Movement Scale (AIMS) or the modified Hillside Simpson Dyskinesia Scale (HSDS) for the 48 patients recruited from the Hillside Hospital (Schooler and Kane, 1982; Basile et al., 1999). Thus, presence of TD included at least one moderate rating or at least two mild ratings on the first seven items of the AIMS (Schooler and Kane, 1982). Because of previous findings of a higher rate of TD in patients of African ancestry compared to those of European ancestry, we analyzed our self-reported African (*N* = 30, 11 of which were classified as having TD) and European (*N* = 187, of which 76 were positive for TD) subjects separately (Jeste and Caligiuri, 1993; Solmi et al., 2018). AIMS scores were available for 155 European patients and 26 African patients. Our European sample has over 80% power to detect an odds ratio of 2.07 [α = 0.05, allele frequency = 0.2, additive model; Quanto v1.2.3; (Gauderman and Morrison, 2006)]. This study was carried out in accordance with the recommendations of Tri-Council Policy Statement 2 and Good Clinical Practice. The protocol was approved by the individual institutional research ethics boards. All subjects gave written informed consent in accordance with the Declaration of Helsinki.

### Genotyping and Analysis

We genotyped the *HSPG2* rs2445142 single-nucleotide polymorphism by polymerase chain reaction amplification with the TaqMan genotyping assay C__16231131_10 (Thermo Fisher Scientific) following the manufacturer’s protocol, followed by genotype determination in the ViiA 7 Real-Time PCR System. Ten percent of the genotypes were repeated for quality assurance, and no mismatches were observed.

Tests for deviation from Hardy–Weinberg Equilibrium were conducted using Haploview (Barrett et al., 2005). We conducted logistic regression analysis of TD occurrence and linear regression analysis of log-transformed AIMS scores using the entire sample, including age and sex as covariates (SPSS).

We carried out the meta-analysis using the R package ‘meta’ with the Mantel-Haenszel fixed-effect method. Using search terms “tardive” and “HSPG2” in PubMed, Scopus, and Web of Science, we found 11 studies, of which six were excluded because they represented review articles (Lee and Kang, 2011; MacNeil and Muller, 2016; Lanning et al., 2017; Zai et al., 2018a,b), one was excluded because it was not a gene association study (Seeman and Tinazzi, 2013), one was excluded because the article was in Japanese (Arinami and Inada, 2011), and one was excluded because the underlying diagnosis was not specified (Bakker et al., 2012). We were able to obtain allele counts for TD cases and controls for the meta-analysis from two studies (Syu et al., 2010; Greenbaum et al., 2012). For the Greenbaum et al. (2012) study, there was also genotype data on rs878949, which is a proxy marker for rs2445142 in a sub-group of the CATIE sample. We included our TD samples (Canada-European, United States-European, Canada/United States-African American, and PGX-European) in the meta-analysis (**Table 1**).

**Table 1:** Demographic information on the samples included in the meta-analysis.

Study/sample	N (TD cases/TD-negative controls)	Ethnicity	Mean age (years)	Males/females
Syu et al. (2010)	86/186	Japanese	56.57	164/110
Greenbaum et al. (2012) Israeli	73/91	Jewish	48.7	89/75
Greenbaum et al. (2012) CATIE^∗^	75/101	European	41.5	145/31
Zai et al. (2013) Canada^∗∗^	40/52	European	42.48	59/33
Zai et al. (2013) United States^∗∗^	24/31	European	34.75	39/16
Zai et al. (2013) Canada/United States^∗∗^	6/13	African American	29.74	15/4
Herbert et al. (2017) IMPACT^∗∗^	20/41	European	41.2	46/15

*^∗^For the CATIE sample, rs878949 genotypes were used as proxies for rs2445142 genotypes. ^∗∗^Previously unpublished data on HSPG2 rs2445142.*

## Results

The genotypes for *HSPG2* rs2445142 did not deviate significantly from Hardy–Weinberg Equilibrium (*p* > 0.05). Our analysis of *HSPG2* rs2445142 in our samples did not yield significant findings with TD occurrence or severity as measured by AIMS (*p* > 0.05). In the CAMH European sample, the marker was not associated with log-transformed total AIMS scores (*t* = 0.162, *p* = 0.872) or TD occurrence [Odds Ratio (adjusted for sex and age) = 1.08, 95% confidence interval: 0.61–1.89; Wald = 0.028; *p* = 0.868]. Similarly, in the CAMH IMPACT sample, the marker was not associated with log-transformed total AIMS scores (*t* = 1.122, *p* = 0.267) or TD occurrence [Odds Ratio (adjusted for sex and age) = 1.22, 95% confidence interval: 0.47–3.17; Wald = 0.168; *p* = 0.682].

We performed a meta-analysis of *HSPG2* rs2445142 in TD occurrence including the Syu et al. (2010) discovery sample, Greenbaum et al. (2012) Israeli and selected CATIE samples, and our Canada (European), United States (European), Canada/United States (African American), and IMPACT (European) samples, totaling 324 TD cases and 515 TD-negative controls. The G-allele was significantly associated with TD [fixed-effects model: OR(G) = 1.51, 95% confidence interval: 1.22–1.86; *p* = 0.0001; Heterogeneity *p* = 0.23], and no significant heterogeneity was observed among the studies included in the meta-analysis (**Table 2**). In addition, the test for Funnel plot asymmetry indicated that the meta-analysis did not suffer from significant publication bias (*p* = 0.116). Meta-regression analyses found age to have a significant effect on our findings [Q(df = 1) = 7.62; *p* = 0.006], while sex appeared to have a trend effect [Q(df = 1) = 3.18; *p* = 0.075]. Results from the sensitivity analysis showed that the Syu et al. (2010) dataset contributed to most of the association signal as well as heterogeneity (**Table 3**). Nonetheless, the direction of effect remained the same throughout the sensitivity analysis.

**Table 2:** Results from meta-analysis of HSPG2 rs2445142 with TD occurrence.

Study	Odds ratio	95%-Confidence interval	%Weight (fixed)	%Weight (random)
Syu et al. (2010)	2.249	1.545–3.272	25.5	26.1
Greenbaum et al. (2012) Israeli	1.599	0.981–2.607	18.2	19
Greenbaum et al. (2012) CATIE	1.232	0.791–1.918	25.1	21.5
Zai et al. (2013) Canada^∗^	1.035	0.535–2.003	12.2	12.3
Zai et al. (2013) United States^∗^	1.073	0.471–2.446	7.7	8.5
Zai et al. (2013) Canada/United States^∗^	0.857	0.218–3.371	3.1	3.4
Herbert et al. (2017) IMPACT^∗^	1.189	0.541–2.611	8.1	9.2
Fixed-effects model^a^	1.507	1.220–1.861	100	
Random-effects model^b^	1.442	1.111–1.873		100

*^a^Fixed-effects model p = 0.0001; ^b^random-effects model p = 0.006. ^∗^Previously unpublished data on HSPG2 rs2445142.*

**Table 3:** Results from sensitivity analysis under fixed-effects model.

Sample omitted	Odds ratio	95%-Confidence interval	*p*-Value	Tau^2^	I^2^ (%)
Syu et al. (2010)	1.2525	0.970–1.618	0.0847	0	0.00
Greenbaum et al. (2012) Israeli	1.4863	1.176–1.878	0.0009	0.0596	37.90
Greenbaum et al. (2012) CATIE	1.5987	1.257–2.033	0.0001	0.0428	29.40
Zai et al. (2013) Canada	1.5726	1.259–1.965	<0.0001	0.0299	25.70
Zai et al. (2013) United States	1.5431	1.241–1.920	<0.0001	0.0396	32.60
Zai et al. (2013) Canada/United States	1.5278	1.234–1.892	0.0001	0.0376	32.90
Herbert et al. (2017) IMPACT	1.5349	1.233–1.911	0.0001	0.0453	35.40

## Discussion

We have conducted an association study and meta-analysis of the *HSPG2* rs2445142 marker in TD occurrence. The finding of the G allele being associated with risk for TD supports a role of this marker in TD, though similar to the meta-analysis of *DRD2* rs1800497 in TD, the effect size of 1.507 was not substantial, thus supporting the notion that TD risk reflects multiple genetic factors.

There are a number of points to consider for the present study. First, the persistence of TD case and TD control status was only assessed in two of the seven included samples (Syu et al., 2010; Greenbaum et al., 2012). Longer term longitudinal observations that include examinations of the fluctuation patterns of TD may help strengthen the genetic findings. In addition, results from the sensitivity analysis indicated that most of the signal may be coming from the discovery sample (Syu et al., 2010), and the observed effect may diminish with subsequent studies. Thus, international efforts are need to provide additional independent replications in large samples, especially for genetic associations with small effect sizes. Moreover, the minor allele frequencies differed across ethnicities, and findings may also be more relevant for East Asian samples in which the original findings were found. Replication studies on patients of various ethnicities may provide insight into whether the genetic association is stronger in East Asians than other populations.

Mutations in the *HSPG2* gene have been observed in patients with Schwartz-Jampel syndrome (chondrodystrophic myotonia), which is an autosomal recessive disorder characterized by bone dysplasia and myotonia (Nicole et al., 2000; Arikawa-Hirasawa et al., 2002a; Stum et al., 2006). This association was supported in mice with reduced expression of perlecan (Arikawa-Hirasawa et al., 1999; Rodgers et al., 2007; Stum et al., 2008). In addition, somatic mutations in *HSPG2* have also been associated with aging of skeletal muscles (Franco et al., 2018), which are coated by a perlecan-containing basement membrane. Perlecan has been found at the neuromuscular junction and is required for acetylcholinesterase clustering at the synapse (Arikawa-Hirasawa et al., 2002b; Guerra et al., 2005; Singhal and Martin, 2011). Because acetylcholinesterase terminates synaptic transmission through the breakdown of acetylcholine, mutations in *HSPG2* may prevent the degradation of acetylcholine, leading to muscle over-excitation (Bordia et al., 2016). Perlecan is also a part of the basement membrane extracellular matrix that makes up part of the blood–brain barrier (Roberts et al., 2012; Marcelo and Bix, 2014). Its C-terminal domain V fragment may play a neuroprotective role following ischemic stroke (Lee et al., 2011). Further work investigating the role of perlecan in neuromuscular junctions and neuroprotection as well as exploring the perlecan-related biological pathways through GWAS approaches will improve our understanding of the potential role this protein plays in TD.

## Author Contributions

CZ, AT, NK, NF, and JK contributed to the design of the project. CZ, FL, JYL, DH, AS, SC, AA, MS, SS, MT, LG, BL, AV, SP, JAL, HM, and GR contributed to data collection. CZ, AT, VdL, GZ, MM, and DM contributed to data analysis. CZ wrote the first draft of the manuscript. All authors reviewed the manuscript.

## Conflict of Interest Statement

JK has received honoraria from Novartis, Roche, and Eli Lilly corporations, and is an unpaid member of the scientific advisory board of AssureRx, Corp. CZ, AT, DM, and JK has patent submissions not related to this submission. HM has received grants or is or was a consultant to: Abbott Labs, ACADIA, Alkemes, Bristol Myers Squibb, DaiNippon Sumitomo, Eli Lilly, EnVivo, Janssen, Otsuka, Pfizer, Roche, Sunovion, and BiolineRx. HM is a shareholder of ACADIA and Glaxo Smith Kline. In the past 3 years JAL reports having received research funding or is a member of the advisory board of Allon, Alkermes, Bioline, GlaxoSmithKline Intracellular Therapies, Lilly, Merck, Novartis, Pfizer, Pierre Fabre, Psychogenics, F. Hoffmann-La Roche, Ltd., Sepracor (Sunovion), and Targacept. JAL receive no direct financial compensation or salary support for participation in these researches, consulting, or advisory board activities. SGP: consultancy/board of advisors/honoraria: American Psychiatric Association, Astra Zeneca, Bristol-Myers Squibb, Cortex, Dainippon-Sumitomo, Janssen Pharmaceutica, Novartis, Otsuka, Pfizer, Roche, Schering Plough, Vanda; research grants: Amgen, Bristol-Myers Squibb, Dainippon Sumitomo, Elan, EnVivo, Forest Laboratories, Janssen Pharmaceutica, Merck, Novartis, Otsuka, Pfizer, Solvay Pharmaceuticals, Roche, Sunovion, NIH, Harvard Massachusetts General Hospital, Brigham and Women’s Hospital, Vanda, speakers’ bureau: Lundbeck, Otsuka, ISCTM, Novartis, Pfizer, Sunovion. In the past 3 years GR has received consultant fees from Laboratorios Farmacéuticos ROVI, Novartis, and Roche, as well as speaker’s fees from Novartis. He holds no commercial investments in any pharmaceutical company. The remaining authors declare that the research was conducted in the absence of any commercial or financial relationships that could be construed as a potential conflict of interest. The reviewer AS and handling Editor declared their shared affiliation at time of review.
